# A Rare Case of Symptomatic Pulmonary Infection Caused by Mycobacterium paraffinicum

**DOI:** 10.7759/cureus.86982

**Published:** 2025-06-29

**Authors:** Huy Vinh, Nirja Sutaria, Arun Adlakha

**Affiliations:** 1 Family Medicine, Edward Via College of Osteopathic Medicine, Spartanburg, USA; 2 Internal Medicine, Atrium Health, Charlotte, USA; 3 Pulmonology, Carolina Lung Clinic, Piedmont Medical Center, Rock Hill, USA

**Keywords:** acid-fast bacilli (afb), chronic obstructive pulmonary disease, copd exacerbation, mycobacterium paraffinicum, non-tuberculous mycobacterium, tree in bud

## Abstract

*Mycobacterium paraffinicum* *(M. paraffinicum) *is a non-tuberculous mycobacterium (NTM) shown to be a human pathogen in recent years. Here, we present a rare case of pulmonary infection caused by *M. paraffinicum *in a 69-year-old female patient who presented with symptoms, initially thought to be from a chronic obstructive pulmonary disease (COPD) exacerbation. From our literature review, this is a case of *M. paraffinicum* presenting as a human pathogen. The patient’s medical history included COPD, obstructive sleep apnea (OSA), and obesity. Her medical management included inhaled corticosteroid (ICS)/ long-acting beta-agonist (LABA), long-acting muscarinic antagonist (LAMA), and nocturnal continuous positive airway pressure (CPAP) device. For her acute worsening of symptoms, she was treated with oral ciprofloxacin and prednisone for possible COPD exacerbation from presumed pneumonia. However, she showed no improvement in her pulmonary symptoms. Follow-up computed tomography (CT) imaging of the chest showed new scattered “Tree in Bud” opacities. Sputum studies and acid-fast bacilli (AFB) cultures revealed the growth of *M. paraffinicum.* She was treated with a combination of oral clarithromycin, moxifloxacin, and rifabutin. This case highlights the importance of keeping atypical mycobacteria, such as NTM, as a cause of pulmonary infection in the differential diagnosis, particularly in patients with chronic pulmonary conditions who present with persistent and/or unexplained respiratory symptoms. Early identification is crucial for appropriate treatment to improve patient outcomes.

## Introduction

Non-tuberculous mycobacteria (NTM) include more than 160 bacterial species, some of which are human pathogens [1]. The most common infections caused by NTM are pulmonary infections [2]. These are most commonly caused by the species *Mycobacterium avium complex* (MAC), *Mycobacterium kansasii*, and *Mycobacterium abscessus complex *[2]. Patients with NTM pulmonary disease typically present with fatigue, fever, weight loss, asthenia, and anorexia. Respiratory symptoms include cough, sputum production, hemoptysis, and dyspnea [1]. High-resolution computed tomography (CT) imaging is required for definitive diagnosis and can show the extent of parenchymal lung damage [1]. Nodular bronchiectasis or small cavitary lesions can be visualized as well, which are commonly seen in MAC-associated pulmonary disease [1]. Humans are frequently in contact with NTM as the bacteria live in soil or any water systems. 

*Mycobacterium paraffinicum* (*M. paraffinicum*) was initially isolated from a soil sample in 1956 [3]. However, in 1971, it lost its standing as a unique species, as it could not be differentiated from *Mycobacterium scrofula*. Several years later, reports determined that *M. paraffinicum* had a unique biochemical response, and after multiple molecular sequence analyses comparing different mycobacterium species, the name *M. paraffinicum* achieved distinct species status in 2010 [4]. 

There are limited reports of *M. paraffinicum* in the clinical setting. Only one report was found of this organism involved in a pseudo-outbreak, at a university-affiliated tertiary care facility in 2009 [4]. This slow-growing, non-tuberculous species was isolated from 21 patients and an ice machine, in a single patient care unit over a 2.5-year period. The hospital's water system was identified as the source of contamination [5]. There have only been three cases published with NTM pulmonary disease caused by *M. paraffinicum*. Both patients in cases reported in 2014 and 2017 were treated with similar anti-mycobacterial antibiotics. However, treatment was discontinued early in both due to intolerable side effects, including nausea and vomiting [4,6]. Another case was reported in 2024, in which the initial clinical impression was pulmonary tuberculosis and follow-up testing led to the identification of *M. paraffinicum*. The patient was treated with anti-mycobacterial antibiotic regimens, with good tolerance and great results [7]. A fourth case of *M. paraffinicum* as a human pathogen was reported in 2019, in a middle-aged male patient with chronic immunosuppression, secondary to immunosuppressive therapy for rheumatoid arthritis (RA). This was the first case of *M. paraffinicum* causing lymphadenitis. Initially, the patient was meant to be treated with rifabutin, clarithromycin, and moxifloxacin. However, the patient’s lymphadenitis resolved with a spontaneous discharge from the involved lymph nodes, before drug therapy was initiated. He also had chronic pulmonary symptoms, but it is unclear if this was caused by NTM, as the sputum cultures were negative [8]. Due to the limited reports of *M. paraffinicum* causing human diseases, little is known about its pathogenic potential, drug susceptibility profile, and treatment outcomes [8].

## Case presentation

**Figure 1:**
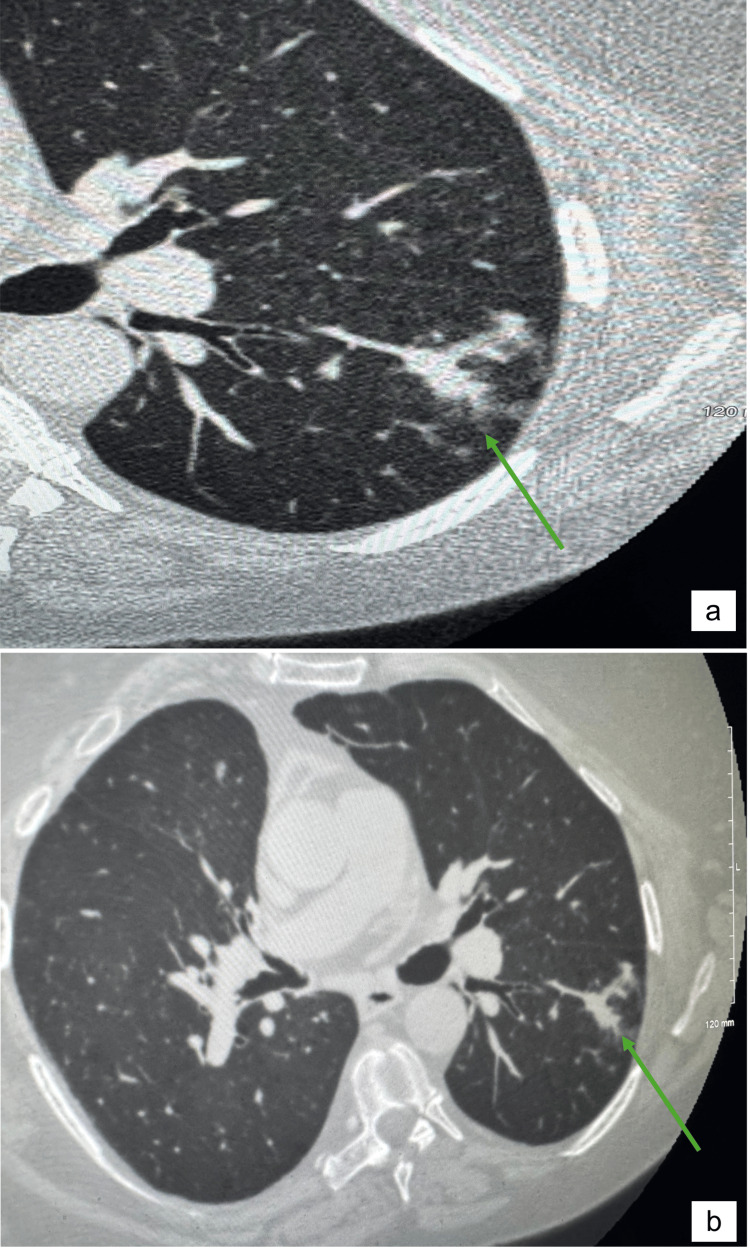
(a, b) CT chest with coned-down view taken on 12/16/19 shows irregular focal opacity in the superior segment of the left lower lobe of the lung (green arrows) CT: computed tomography

In December 2019, a 69-year-old female patient presented to the Carolina Lung Clinic with symptoms of chronic obstructive pulmonary disease (COPD) exacerbation, such as an increased productive cough with white sputum. She had been followed in the clinic for several years due to her past medical history of obesity (BMI 45 kg/m^2^), obstructive sleep apnea, COPD, and right lower lung lobectomy for a benign pulmonary process in 2007. Her home medications included an inhaled corticosteroid (ICS)/long-acting beta-agonist (LABA), a long-acting muscarinic antagonist (LAMA), and nocturnal continuous positive airway pressure (CPAP). She was advised weight loss and pulmonary rehabilitation. When she presented to the clinic with shortness of breath and increased sputum production, a chest X-ray followed by a CT chest was performed, showing irregular focal opacities in the superior segment of the left lower lobe (Figure 1).

Findings also included emphysematous changes in the upper lobes, evidence of prior granulomatous disease, and postsurgical changes consistent with a previous right lung lobectomy.

**Figure 2:**
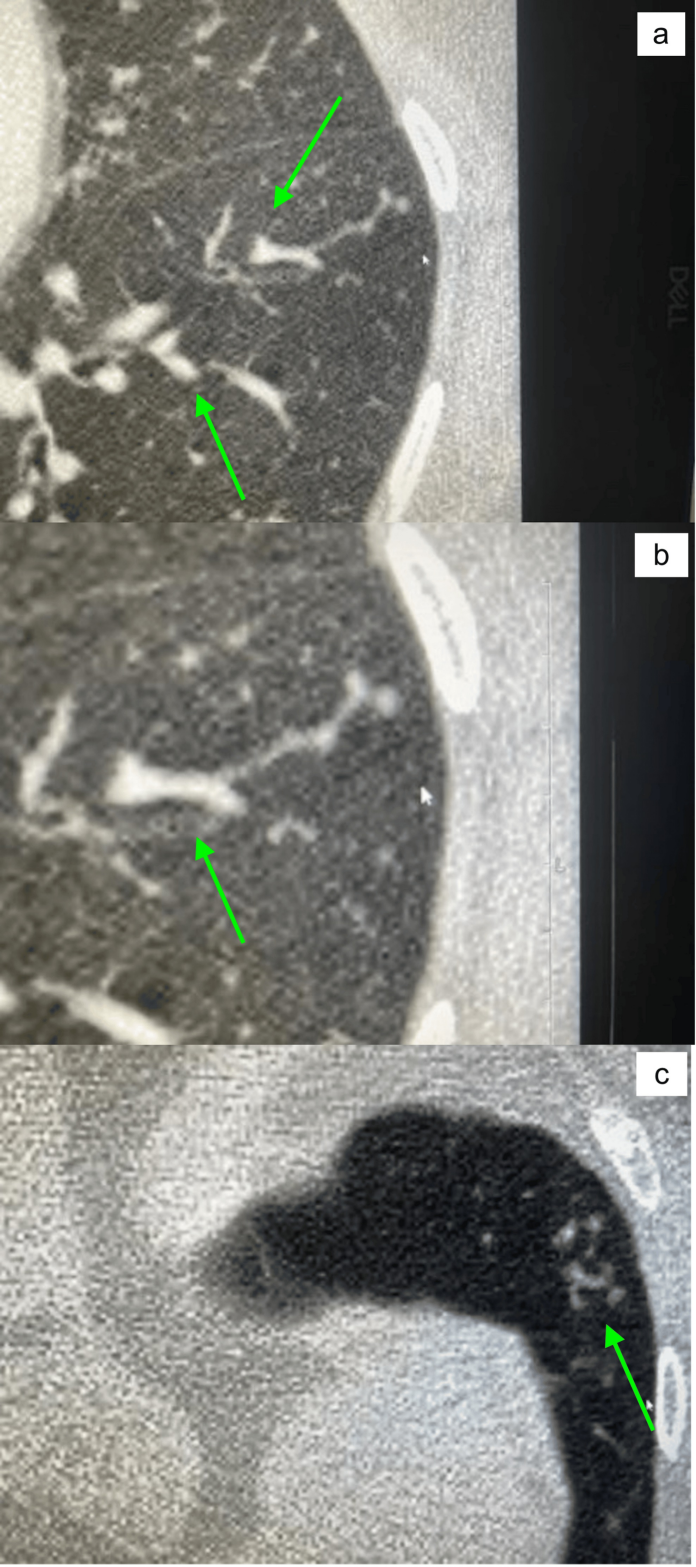
(a-c) CT chest taken on 03/17/20 showing new scattered areas of “Tree in Bud” opacities (green arrows) within the entire left lung CT: computed tomography

The patient was presumed to have community-acquired pneumonia and was empirically treated with oral ciprofloxacin 500 mg twice daily and prednisone 20 mg daily for 14 days. She improved clinically with these medications until her next COPD flare-up in February 2020, where she was again treated with the same combination of medications. During this exacerbation, the patient continued to have residual pulmonary symptoms, and a follow-up CT chest was done the following month. Results showed that the left lower lobe irregular focal opacities had resolved. However, new scattered “Tree in Bud” opacities were noted in the entire left lung (Figure 2).

These changes were suspicious for an atypical infection, including atypical mycobacteria. The rest of her lung findings remained the same as before.

Multiple sputum studies were ordered to check for bacterial cultures, acid-fast bacilli (AFB), and fungal organisms. The cultures showed normal respiratory flora with an abundant quantity of beta-lactamase negative* Hemophilus parainfluenza*. The patient was treated with oral amoxicillin/clavulanic acid (Augmentin, US Antibiotics) 875 mg twice daily for 14 days, which was the drug of choice for the treatment of this organism. Her sputum also grew *Candida albicans*, for which she was prescribed 14 days of oral fluconazole (Diflucan, Pfizer Medical). Her final AFB cultures on 4/27/20 (Table 1) grew *M. paraffinicum*.

**Table 1:** Final report of the AFB sputum studies on 04/27/20 AFB: Acid-fast bacilli

Species	Results
Mycobacterium tuberculosis complex	Negative
Mycobacterium avium complex	Negative
Mycobacterium paraffinicum	Positive

**Figure 3:**
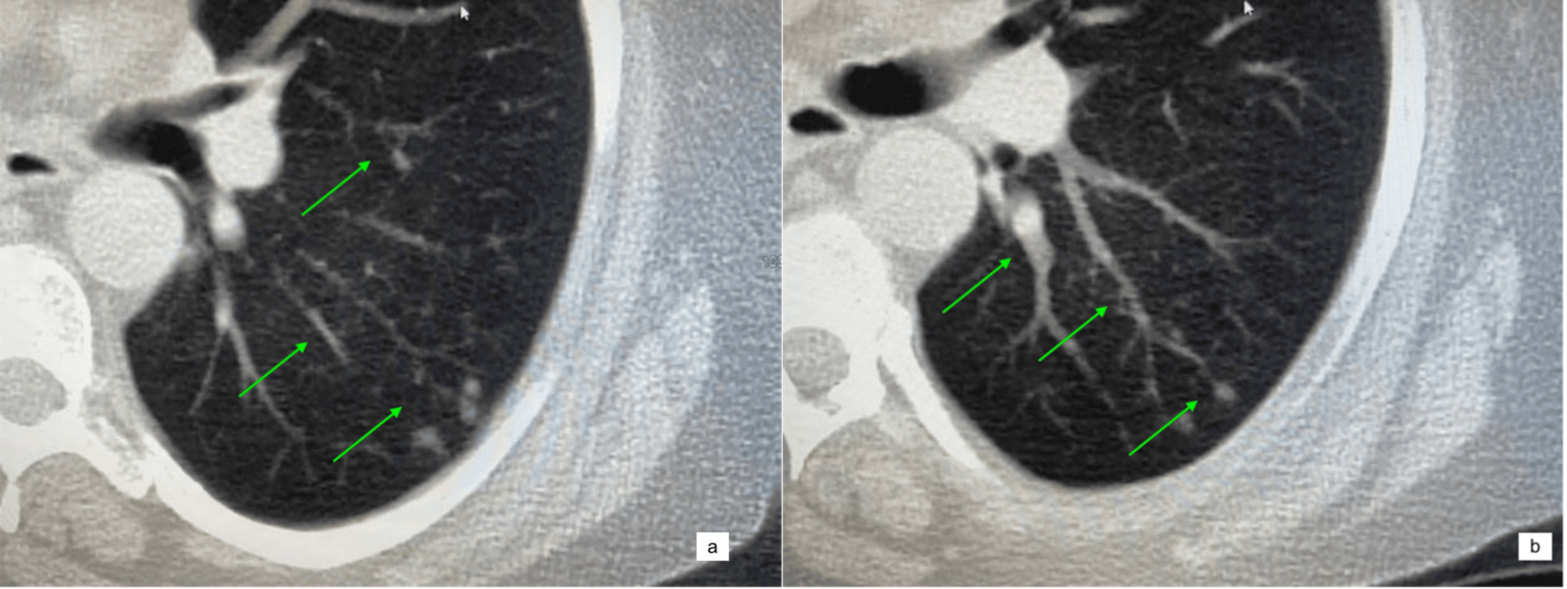
(a, b) CT chest taken on 05/26/21 showing an increased cluster of micronodules in the left base (green arrows) CT: computed tomography

The patient was then referred to an infectious disease specialist due to the presence of new “Tree in Bud” opacities in her CT chest and the isolation of NTM. Repeat sputum AFBs and CT chest scan were ordered. The repeat sputum studies were intermittently positive for *M. paraffinicum*, and the CT chest showed waxing and waning of radiographic findings. At baseline, the patient’s symptoms were stable and there were no significant constitutional symptoms. She was continued on all her respiratory medications, including pulmonary hygiene, and was followed very closely. A repeat CT scan of the chest on 5/26/21 (Figure 3) showed a progression of the disease with an increased cluster of micronodules in the left base, and stable nodules scattered throughout the remainder of the lungs.

**Figure 4:**
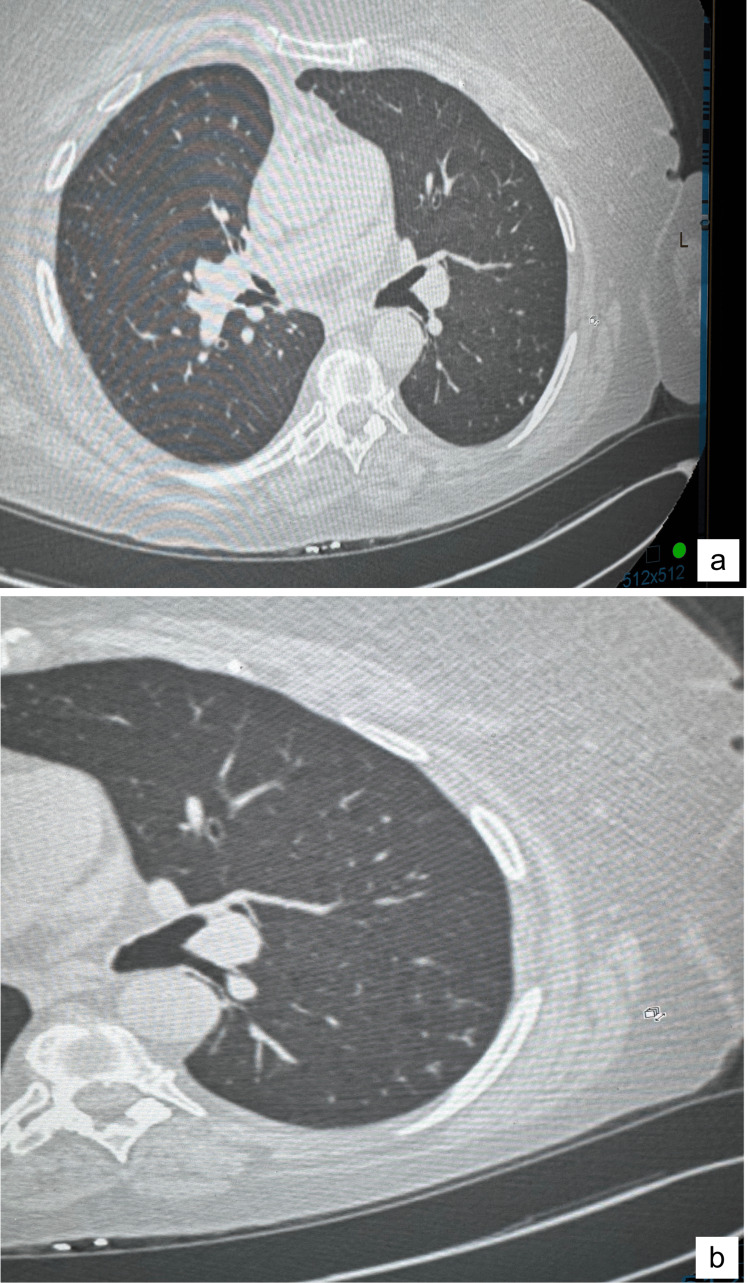
CT chest with coned-down view taken on 09/20/22 showing resolution of the cluster of micronodules and nodules in the left lung base after treatment CT: computed tomography.

Per an infectious disease specialist’s recommendations, she was initiated on triple drug therapy with clarithromycin 500 mg twice daily, moxifloxacin 400 mg daily, and rifabutin 300 mg daily. She had regular clinical follow-ups with sputum AFB cultures ordered monthly. The anticipated duration of treatment was 12 months from the date of the last negative sputum AFB study. Overall she required nearly 18 months of anti-mycobacterial antibiotic therapy. Both her symptoms and CT scans of chest improved significantly with therapy (Figure 4).

Changes of the emphysema in the upper lobes and the old granulomatous lung disease were stable.

## Discussion

NTM are important human pathogens that have posed great diagnostic and management challenges over the years due to the limited studies and data on in vitro susceptibility. Patients with severe and/or progressive NTM pulmonary disease are considered candidates for anti-mycobacterial multidrug therapy, especially those with nodular bronchiectasis disease, like our case [1]. However, as there are limited cases and treatment outcomes published about NTM pulmonary disease, most recommendations for therapy are based on retrospective cohort studies, drug susceptibility surveys, or animal experiments [1].

Four case reports have been published to date with* M. paraffinicum* causing disease in humans, with only three cases involving the lungs. One was in a middle-aged man, where the organism was shown to cause lymphadenitis. The other three were pulmonary infections in the elderly with advanced bronchiectasis at presentation: one with nodular bronchiectasis and two with the chronic cavitary form. These presentations were very similar to our patient, as she was an elderly female with a chronic pulmonary history, and was found to have nodular bronchiectasis with “Tree in Bud” opacities and scattered lung nodules on CT imaging. As *M. paraffinicum* was recently identified as a unique species, there were no previous susceptibility data to guide therapy in the first reported case. Therefore, an empiric anti-mycobacterial antibiotic regimen was prescribed to the first patient, who followed an oral regimen against slow-growing mycobacteria: azithromycin, ciprofloxacin, and linezolid. The second patient was treated with rifabutin, clarithromycin, and ciprofloxacin. Both patients were unable to tolerate their treatments due to gastrointestinal complaints. The third patient was treated with oral rifampicin, ethambutol, azithromycin, and intravenous amikacin [4-7]. Our patient was treated with clarithromycin, moxifloxacin, and rifabutin with close monitoring of pulmonary symptoms, sputum AFB, and drug-related side effects. The anticipated duration of treatment was 12 months from the date of the last negative sputum AFB study. She required nearly 18 months of drug therapy with subjective (symptoms) as well as objective (chest X-ray and CT scan of the chest) improvement.

## Conclusions

This case underscores the potential of *M. paraffinicum *to act as a human pathogen, particularly as a cause of symptomatic pulmonary disease. It highlights the importance of recognizing this atypical mycobacterium as a potential pathogen in patients with unresolved pulmonary symptoms, particularly those exhibiting CT chest abnormalities, such as "Tree in Bud" opacities, with or without nodular or cavitary bronchiectasis. Further studies are necessary to determine the optimal treatment strategies for this newly reinstated NTM species, which will help improve patient outcomes, promote early intervention, and optimize preventive measures.
